# Combined left hepatectomy with fenestration and using a harmonic scalpel, fibrin glue and closed suction drainage to prevent bile leakage and ascites in the management of symptomatic polycystic liver disease: a case report

**DOI:** 10.4076/1752-1947-3-7442

**Published:** 2009-08-27

**Authors:** Christopher Kosmidis, Christopher Efthimiadis, George Anthimidis, Sofia Levva, John Prousalidis, Konstantinos Papapolychroniadis, Epaminondas Fachantidis

**Affiliations:** 1 Department of Surgery, Interbalkan European Medical Center, Thessaloniki, Greece; 2 1st Propedeutic Surgical Clinic, Aristotle University of Thessaloniki, AHEPA Hospital, Greece

## Abstract

**Introduction:**

Surgical treatment is the usual therapy for patients with polycystic liver disease and with severe symptoms, yet the results of surgery are often disappointing and the optimal surgical approach is uncertain.

**Case presentation:**

We present the case of a 41-year-old Greek woman who underwent combined left hepatectomy with fenestration for symptomatic polycystic liver disease using ultrasound scalpel, fibrin glue and closed suction drain to prevent bile leakage, haemorrhage and ascites. Liver resection using the ultrasound scissors allowed quick parenchyma dissection under haemostatic conditions with safe coagulation of small vessels and bile ducts. Moreover, the ultrasound scalpel was applied to the cyst cavities exposed on the peritoneum to ablate the fluid-producing epithelial cyst lining. We also covered the cut cystic cavities exposed to the peritoneum surface of the liver with fibrin glue. Instead of allowing the opened cysts to drain into the abdominal cavity, we used two wide bore closed suction fluted drains. We did not observe excessive fluid loss through the drainage after the second postoperative day. The drain tubes were removed on the third postoperative day.

**Conclusion:**

In our patient, effective treatment of ascites and prevention of bile leakage and bleeding indicate that this new approach is promising and may become a useful surgical technique for polycystic liver disease.

## Introduction

Polycystic liver disease (PLD) is a common manifestation of polycystic kidney disease and is associated with an autosomal dominant inheritance. Patients are usually asymptomatic [1]. Symptomatic PLD has been treated by percutaneous aspiration with or without sclerotherapy, drainage of the superficial cysts into the abdominal cavity and fenestration of deeper cysts into the superficial cyst cavities via laparotomy or laparoscopy, hepatic resection or orthotopic liver transplant. The results of surgery are often disappointing, with quick return of symptoms, bile leakage and symptomatic ascites in many patients [1]-[3].

We present the case of a patient who underwent combined left hepatectomy with fenestration for symptomatic PLD using the harmonic scalpel, fibrin glue and closed suction drain to prevent bile leakage and ascites.

## Case presentation

**Figure 1:**
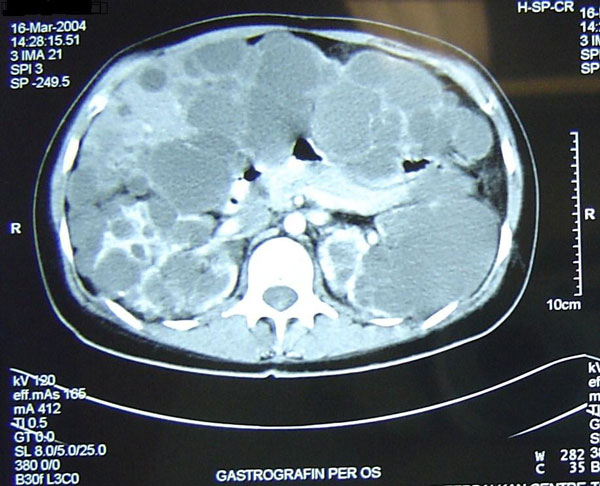
**Pre-operative computed tomography demonstrating multiple cysts in the liver, anterior surface of the pancreas and kidneys**.

A 41-year-old Greek woman presented with chronic and unrelenting right upper quadrant pain, epigastric fullness, early satiety, nausea, vomiting and dysphagia. On physical examination, hepatomegaly and tenderness in the right upper quadrant were found. Abdominal ultrasound (US), computed tomography (CT), magnetic resonance imaging (MRI) and magnetic resonance cholangiopancreatography (MRCP) revealed multiple liver cysts, particularly in the left hepatic lobe, with characteristics similar to simple hepatic cysts. Multiple cysts were also found in the kidneys and the anterior surface of the pancreas (Figure 1). The left hepatic lobe was enlarged, compressing the stomach to the spleen. Serum biochemical analysis demonstrated a mild impairment of liver function: serum glutamic oxaloacetic transaminase (SGOT) 126 U/L (10-31 U/L), Serum glutamic pyruvic transaminase (SGPT) 85 U/L (10-31 U/L), while urea and creatinine were within the normal range.

The patient's family history was positive for the presence of PLD. Her 63-year-old mother had multiple non-parasitic asymptomatic cysts in the liver and kidneys. Additionally, her 17-year-old daughter and 13-year-old son had multiple cysts in the kidneys, while the liver, the pancreas and the spleen were normal. Given the family history and the presence of multiple cysts in the liver, kidneys and the anterior surface of the pancreas, the diagnosis of PLD associated with polycystic kidney disease was made.

**Figure 2:**
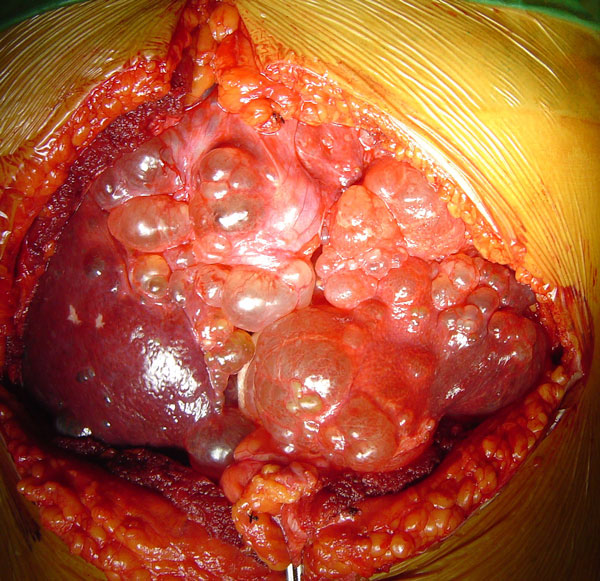
**Intra-operative view of multiple small cysts all over the left hepatic lobe and one of the three large dominant cysts located in the right hepatic lobe**.

**Figure 3:**
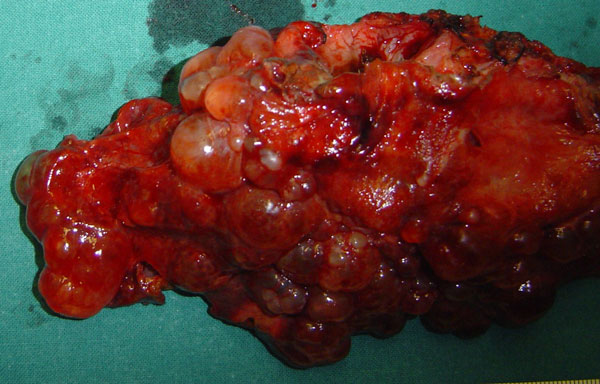
**The specimen from the left hepatic lobe**.

A double Kocher incision was made to provide excellent access to the upper abdomen. The left hepatic lobe was enlarged and full of multiple cysts, the maximum diameter of which was 9 cm. The stomach was compressed between the left lobe of the liver and the spleen, explaining the cyst-related complaints of the patient. Furthermore, multiple small cysts and three large dominant cysts (diameter: 7-13 cm) were located at the right hepatic lobe (Figure 2). There were huge cysts throughout both kidneys and small cysts at the anterior surface of the pancreas. The hepatoduodenal ligament was exposed to provide access for vascular clamping and identification of major vascular and biliary structures. The liver was mobilized by the division of hepatic peritoneal attachments. A left hepatic lobectomy, that is of segments II, III, IV, was made using ultrasound scissors (Harmonic Scalpel, UltraCision, Ethicon Endosurgery, Cincinnati, Ohio, USA). The diameter of the removed lobe was 17 cm (Figure 3). Cysts located on the surface of the right hepatic lobe, including the three dominant cysts, were surgically unroofed and windows were created by fenestration between superficial cysts and adjacent deep cysts. The fluid from the opened cysts was rapidly aspirated under continuous suction.

After completion of the resection, the tourniquet was opened and the remaining cut surface carefully inspected for residual bleeding or nonoccluded bile ducts. The cut surface was plain and brownish; biliary leaks or persistent bleeding were easily detected and sutured with 4-0 polypropylene. The ultrasound scalpel was applied to the cyst cavities exposed on the peritoneum in order to attempt ablation of the fluid-producing epithelial cyst lining. To avoid bile leakage and haemorrhage, fibrin glue was spread over the raw surface of the liver. Cysts in the pancreas and kidneys were not treated. Two wide-bore closed suction fluted drains (30 F) were brought out through a separate stab wound; one placed in the subhepatic space and one in the right subdiaphragmatic space. Postoperatively, the patient remained well and without complications. The drain tubes were removed on the third postoperative day, when the drainage had decreased to less than 30 mL in 8 hours. Symptomatic relief and reduction in abdominal girth were achieved. Histologic examination showed von Meyenburg's complexes. The patient was followed up at clinic - special data included hepatic and renal function, symptomatic relief, the patient's working capacity and CT scans. The follow-up showed post-resection hypertrophy of the spared liver and lack of clinically significant cyst progression. Four years after the procedure, the patient had an improved quality of life and functional status without deterioration in her hepatic or renal function.

## Discussion

With the widespread use of sensitive imaging techniques, the frequency of non-parasitic hepatic cysts is reported more often. Adult polycystic disease is the most common cystic disease. Liver cysts in patients with polycystic kidney disease are generally asymptomatic, but in a few patients, hepatomegaly from numerous large cysts may cause symptoms [1,4].

Treatment is usually only carried out in patients with severe symptoms related to large cysts or complications. Associated medical problems, especially intracranial aneurysms and valvular heart disease need to be evaluated in patients with PLD [1,2]. Surgical management differs from that for patients with simple cysts or cystadenomas because multiple cysts continue to grow and appear *de novo* after treatment [5]. Therefore, the therapeutic aim is to significantly reduce the size of the polycystic liver without compromising liver function, and to provide long-term relief of symptoms. The optimal treatment for symptomatic PLD is uncertain. There is no clear consensus regarding the optimum timing of intervention and the surgical approach is based in part on the number, size and location of the cysts. The surgical therapy should be tailored to the extent of disease in each patient.

In our case, the patient was classified as Type II, based on Gigot's classification, that is, diffuse involvement of liver parenchyma by medium-sized cysts with remaining large areas of non-cystic parenchyma [3]. Therefore, the combination of hepatic resection with fenestration appeared to be a valuable option, allowing for the removal of multiple segments grossly affected (II, III, IV) and reduction in liver mass with maximal preservation of liver parenchyma. Fenestration alone would probably be unsuccessful because the liver parenchyma might be more rigid due to the fibrosis around the cysts, and the cysts would not collapse as expected after fenestration. Likewise, the large superficial and deep-seated cysts within the right hepatic lobe with more normal parenchyma should undergo fenestration.

The most commonly reported morbidities with combined fenestration and resection are pleural effusions, wound infection, ascites, transient biliary leaks and bleeding [6]-[10]. The surgical technique is an important factor in preventing intra-operative and postoperative complications. Various techniques have been developed for safe and careful dissection of the liver parenchyma [9,10]. The high number of techniques used worldwide shows the lack of a generally accepted gold standard. Technical improvement seems to be possible and desirable. The aim of our study was to prove the suitability of the ultrasound scissors, closed suction drain and fibrin glue in surgery for PLD. In our clinic, this cutting device is mainly used in laparoscopic surgery for dissection of tissue, but we consider it an appropriate instrument for liver dissection. Because of its simultaneous haemostatic and coagulating effect, it might theoretically offer a considerable advantage in surgery for PLD [10].

Handling of the instrument, cutting and coagulation quality were satisfactory and safe. To achieve a better and more effective coagulating effect, the portal structures were occluded by a tourniquet which did not last longer than 30 minutes, together with lowering of the central venous pressure during resection. The liver resection using the ultrasound scissors allowed quick parenchyma dissection under haemostatic conditions with safe coagulation of small vessels and bile ducts of up to 2 to 3 mm in diameter. Larger vessels and biliary ducts were divided with clamps and sutured with 4-0 polypropylene. The major advantage of the ultrasound scalpel was the modest trauma that it produced and the controlled dissection of the tissue. Especially in the periphery, the UltraCision was an ideal dissection instrument: with the absence of large vessels and bile ducts, nearly all of the parenchyma was easily divided without causing bleeding, bile leakage or trauma. Moreover, use of ultrasound scissors on the cyst cavities exposed on the peritoneum was attempted to facilitate ablation of the secretory epithelium and reduction of postoperative continual peritoneal fluid losses. However, we also covered the cut cystic cavities exposed to the peritoneum surface of the liver with fibrin glue [11,12]. Fibrin glue causes less intra-abdominal adhesions while allowing shorter haemostasis time than primary suture [13]. Moreover, instead of allowing the opened cysts to drain into the abdominal cavity, two wide-bore closed suction fluted drains were used.

Ascites is the commonest complication specific to surgery for PLD, occurring in all patients undergoing resection [1,2]. We at no time encountered excessive fluid loss through the drainage after the second postoperative day. Effective treatment of ascites in our patient may be related to some extent to the use of the UltraCision instrument as well as the particular type of drain, which prevents accumulation of ascitic fluid and ensures complete evacuation of the collection and collapse of the opened cystic cavities. However, other factors may play a major role, such as the use of fibrin glue to seal the cut liver surface or the type of surgery.

## Conclusions

Surgical management of patients with PLD remains a challenging issue for physicians. The aim of the present study was to investigate the ability of the UltraCision instrument, fibrin glue and closed suction drainage in hepatic resection combined with fenestration for PLD. This method appears to be an advantageous new technique. This case report is not sufficient to draw any final conclusions. Therefore, the benefits of this surgical approach should be further evaluated. However, our initial experience is promising, and we believe that it may become a valuable means in surgery for PLD.

## Abbreviations

CT: computed tomography; MRCP: magnetic resonance cholangiopancreatography; MRI: magnetic resonance imaging; PLD: polycystic liver disease; SGOT: serum glutamic oxaloacetic transaminase; SGPT: serum glutamic pyruvic transaminase; US: ultrasound.

## Consent

Written informed consent was obtained from the patient for publication of this case report and any accompanying images. A copy of the written consent is available for review by the Editor-in-Chief of this journal.

## Competing interests

The authors declare that they have no competing interests.

## Authors' contributions

CK, CE and EF performed the operation and together with GA and SL contributed to the conception and design of the manuscript. JP and KP analyzed and interpreted the patient regarding the polycystic disease. GA and SL were major contributors in writing the manuscript. All authors read and approved the final manuscript.
